# PROTOCOL: Effects of vitamin and mineral supplementation during pregnancy on maternal, birth, child health and development outcomes in low‐ and middle‐income countries: a systematic review

**DOI:** 10.1002/CL2.191

**Published:** 2018-11-22

**Authors:** Emily C Keats, Aamer Imdad, Zulfiqar A Bhutta

## Background

### The problem, condition or issue

Close to 2 billion people today are deficient in key vitamins and minerals; of these individuals, the vast majority are women and children residing in low‐ and middle‐income countries (LMICs) [Black 2013; Darnton‐Hill 2015]. Among women of reproductive age (WRA) in LMICs, micronutrient deficiencies result from diets that chronically lack diversity and thus do not provide sufficient amounts of essential vitamins and minerals to meet recommended daily allowances [FAO and WHO 2004]. In some cases, infections and/or chronic disease may contribute to micronutrient deficiencies by directly inhibiting nutrient absorption [Bailey 2015].

Because of increased nutritional requirements throughout pregnancy, micronutrient deficiencies are often exacerbated during this time. Additionally, repeated pregnancies and short inter‐pregnancy intervals have been shown to contribute to poor maternal micronutrient status [Darnton‐Hill 2015]. As such, multiple concurrent deficiencies (in two or more micronutrients) are common among pregnant women, especially in LMICs [Black 2003; Allen 2009]. Though population‐level estimates are mostly lacking, the global prevalence of prenatal iron deficiency anaemia is estimated to be 19.2% (95% confidence interval (CI) 17.1%‐21.5%), while vitamin A deficiency affects 15.3% (95% CI 6.0%‐24.6%) of pregnant women [Black 2013]. Restricting to LMICs could produce slightly larger prevalence estimates.

Micronutrient deficiencies are associated with a host of adverse outcomes for both the mother and the baby. Anemia in pregnancy, typically caused by iron deficiency, increases the risk of maternal mortality, perinatal mortality, and low birthweight [Haider 2013; Christian 2010; Allen 2001]. Low folate levels are unequivocally associated with neural tube defects (NTD) [De‐Regil 2015], and severe iodine deficiency affects fetal development, including increasing the risk of mental retardation and cretinism [Dunn 1993]. Low calcium intake during pregnancy is associated with the development of hypertension, and hypertension is one of the leading causes of maternal morbidity, mortality, fetal growth restriction and preterm birth [Ortega 1999; Bucher 1996; Hofmeyr 2006]. Similarly, low vitamin D levels throughout gestation can lead to pre‐eclampsia and, subsequently, increase the risk of preterm birth, small‐for‐gestational age (SGA), and perinatal mortality [Dror 2011; MacKay 2001; De‐Regil 2016]. The effects of maternal zinc deficiency are not well understood, but it has been suggested that zinc supplementation during pregnancy can result in the reduction of preterm birth [Ota 2015]. Maternal malnutrition has also been shown to manifest through intergenerational effects, impacting the short‐term and long‐term outcomes of offspring, including growth, neurodevelopment and cognition, and cardiometabolic, pulmonary, and immune function [Gernand 2016]. Poor maternal nutrition reduces a newborn's chance to achieve proper growth and development in the short‐term and, together, these early life inputs can establish the trajectory for chronic and other diseases later in life. Evidence has indicated that poor fetal and infant growth can lead to stunting in adulthood, chronic diseases relating to nutrition, lower educational attainment, reduced income, and even decreased birthweight in the next generation [Victora 2008], highlighting the immense health and socail consequences of maternal manutrition.

### Description of the intervention

Several strategies exist for reducing micronutrient malnutrition among women. These include diet diversification, large‐scale and targeted fortification, biofortification of staple crops, and micronutrient supplementation with tablets or powders [Bhutta 2008]. This review will encompass micronutrient supplementation interventions during pregnancy.

Generally, micronutrient supplementation is used as a short term, preventive strategy that is targeted towards specific at‐risk population groups [Bailey 2015]. As such, supplementation has been recommended as part of routine antenatal care to overcome the complications associated with micronutrient deficiencies during pregnancy.

Within the context of routine antenatal care for pregnant women, the World Health Organization (WHO) currently recommends daily iron folic acid supplementation with 30‐60 mg of elemental iron and 400 μg folic acid [WHO 2016]. In populations where anaemia prevalence is less than 20% or where side effects from daily supplementation are severe, weekly iron folic acid supplementation with 120 mg of elemental iron and 2800 μg folic acid is recommended instead [WHO 2016]. The WHO has issued several context‐specific recommendations as well: i) daily calcium supplementation (1.5‐2.9 grams oral elemental calcium) in populations with low dietary intake of calcium; and ii) daily (up to 10,000 IU) or weekly (up to 25,000 IU) vitamin A supplementation where vitamin A deficiency is a severe public health problem [WHO 2016]. Currently, zinc supplementation is recommended only where there is rigorous research to support its provision, and vitamin D supplementation is not recommended for pregnant women to improve maternal and perinatal outcomes [WHO 2016].

To address the issue of multiple deficiencies, the United Nation Children's Fund (UNICEF), United Nations University (UNU), and the WHO developed a multiple‐micronutrient (MMN) tablet that provides the daily recommended intake of vitamin A, vitamin B1, vitamin B2, niacin, vitamin B6, vitamin B12, folic acid, vitamin C, vitamin D, vitamin E, copper, selenium, and iodine with 30 mg iron and 15 mg of zinc for pregnant women [UNICEF, WHO, and UNU 1999]. Other such tablets have been developed for supplementation trials on a case‐by‐case basis, typically providing at least three essential micronutrients.

More recently, the use of lipid‐nutrient supplements (LNS) has been proposed to combat the adverse effects of maternal micronutrient deficiencies. Similar to MMN supplements, LNS contain a range of vitamins and minerals, but also provide energy, protein, and essential fatty acids. They are considered lipid‐based because energy from LNS comes in the form of fats, such as vegetable fat, peanut/groundnut paste, milk powder and sugar [Arimond 2015]. Lipid‐based products like Plumpy'nut were traditionally used for the treatment of severe acute malnutrition, but have since been adapted to contain a lower dose of energy such that daily supplementation with LNS products could be used as a preventive therapy for undernutrition [Arimond 2015].

Supplementation with MMN is not recommended for pregnant women to improve maternal and perinatal outcomes, as more research is needed [WHO 2016]. The WHO has not yet issued any guidance for LNS [WHO 2016].

### How the intervention might work

Micronutrients, essential vitamins and minerals that are obtained from the diet, are critical for a host of metabolic activities that support tissue growth and functioning. As such, they are fundamental in enabling the healthy development of the fetus and promoting optimal pregnancy outcomes. Antenatal micronutrient supplementation interventions aim to increase circulating levels of vitamins and minerals in pregnant women in order to meet the recommended daily intakes, which are higher than normal due to increased physiological demands during pregnancy. Through tablets or other vehicles (e.g. syrup, drops, powder, or food matrices), the micronutrients are ingested and bioconverted to their active form in order to support maternal health and fetal development throughout gestation.

Through primary studies and meta‐analysis of randomised controlled trials (RCTs), some antenatal micronutrient supplementation interventions have proven to be efficacious in improving congenital/birth outcomes, including lowering the risk of NTDs, cretinism, premature rupture of membranes (PROM), low birthweight, and preterm birth [Haider 2013; De‐Regil 2015; De‐Regil 2016; Ota 2015; Lassi 2013; Rumbold 2015; Bougma 2013; Zhou 2013]. The duration of exposure needed to produce clinically meaningful results may vary depending on the supplement. For example, it is recommended that folic acid supplementation begins as early as possible, and ideally prior to conception [WHO 2016], while daily iron supplementation that begins mid‐gestation has been effective at improving some outcomes [Peña‐Rosas 2015].

### Why it is important to do the review

There are several existing systematic reviews that examine the impact of single and multiple micronutrient supplementation interventions in pregnancy [Appendix 1], many of which incorporate data from trials conducted in low and middle‐income settings. However, significant heterogeneity in results has been reported (e.g. for antenatal iron supplementation); this has not yet been explained by subgroup analysis. In addition, inconclusive results for several micronutrient supplementation interventions (e.g. folic acid supplementation for maternal health and pregnancy outcomes, calcium supplementation (other than for preventing or treating hypertensive disorders) for pregnancy and infant outcomes, and zinc supplementation for improving pregnancy and birth outcomes) were found, warranting further investigation. Many of the systematic reviews listed [Appendix 1] are several years old, underscoring the need to update the evidence in order to capture newly completed trial data. There is the hope that with more power to detect differences, some unanswered questions will be resolved. For example, additional exploration is required to confirm the sex‐specific differences in infant mortality following antenatal MMN supplementation that was noted in a study by Smith and colleagues [Smith 2017]. Additionally, concerns have been raised regarding the safety of iron supplementation in women with high haemoglobin concentrations, and the potentially negative long‐term consequences that unabsorbed iron may have on child morbidity [Mwangi 2017; Paganini 2016].

In addition to the limitations of existing systematic reviews of RCTs, the effectiveness of antenatal micronutrient supplementation interventions in a real world setting has not been well established.

We aim to understand which antenatal supplementation interventions are effective at improving key maternal and child health, nutrition, and mortality outcomes in LMICs. We will include data from large programme evaluations as well as smaller studies. Additionally, we will include adolescent women as a pre‐specified subgroup, which will help to elucidate strategies that can mitigate the risks associated with adolescent pregnancy in LMICs [Bhutta 2017]. Lastly, we hope to answer some of the remaining questions outlined above, including potential infant sex‐specific differences and safety concerns following supplementation in pregnancy. Taken together, these results will inform the evidence on which to base policy and programming relating to micronutrient supplementation in pregnancy for women in LMICs. In addition, this review will point to any gaps in the existing evidence.

## Objectives

This review will summarize the available evidence on antenatal micronutrient supplementation interventions in LMICs. For each intervention, results will be summarized separately.

Specific objectives:

1. What is the impact of single micronutrient supplementation (calcium, vitamin A, vitamin D, iodine, zinc, vitamin B12) during pregnancy on maternal, birth, child health and development outcomes?
2. What is the impact of iron folic acid supplementation during pregnancy on maternal, birth, child health and development outcomes?
3. What is the impact of multiple micronutrient supplementation during pregnancy on maternal, birth, child health and development outcomes?
4. What is the impact of lipid‐based nutrient supplementation during pregnancy on maternal, birth, child health and development outcomes?

## Methodology

### Criteria for including and excluding studies

#### Types of study designs

We will include the following study designs:

► Randomized controlled trials (RCTs), where participants were randomly assigned, individually or in clusters, to intervention and comparison groups. Cross‐over designs will be eligible for inclusion.
► Quasi‐experimental designs, which include:
  ▷ Natural experiments: studies where non‐random assignment is determined by factors that are out of the control of the investigator. One common type includes allocation based on exogenous geographical variation.
  ▷ Controlled before‐after studies (CBA), in which measures were taken of an experimental group and a comparable control group both before and after the intervention. We also require that appropriate methods were used to control for confounding, such as statistical matching (e.g. propensity score matching, or covariate matching) or regression adjustment (e.g. difference‐in‐differences, instrumental variables).
  ▷ Regression discontinuity designs; here, allocation to intervention/control is based upon a cut‐off score.
  ▷ Interrupted time series (ITS) studies, in which outcomes were measured in the intervention group at least three time points before the intervention and after the intervention.

Reviews will be excluded.

#### Types of participants

Participants will include healthy (i.e. non‐diseased) pregnant women of any age and parity living in LMICs. LMICs will be defined by the World Bank Group at the time of the search for this review. Though our aim is to include healthy pregnant women, the prevalence of micronutrient deficiencies is high in these settings, indicating that women are likely to have one or more micronutrient deficiencies at baseline; women will not be excluded on this basis. Studies that include only a subset of eligible participants will be retained as eligible, but will only be included in analysis where data has been disaggregated appropriately for use.

#### Types of interventions

The following interventions targeting pregnant women will be included, and will be analysed separately:

► Single micronutrient supplementation (calcium, vitamin D, iodine, folic acid, iron, vitamin A, zinc, vitamin B12) compared to placebo
  ▷ Supplementation may take the form of tablets, drops, syrup, or powder
► Iron folic acid supplementation compared to folic acid alone or placebo
► Vitamin D and calcium supplementation compared to placebo
► MMN compared to iron folic acid supplementation or placebo
  ▷ For MMN, trials that use fewer than three micronutrients in its composition will be excluded [Kawai 2011; Haider 2017]
► LNS compared to MMN or placebo

For logistical reasons, we have not included every vitamin and mineral. Interventions were chosen based on relevance (i.e., most prevalent nutritional deficiencies) and data availability when considering the LMIC context.

There will be no restrictions regarding: i) the duration of exposure to the intervention, ii) the provider of the intervention, iii) the frequency of the intervention (e.g. daily or intermittent supplementation), or iv) the food vehicle utilized for LNS interventions. We will include studies where co‐interventions (e.g. education) are provided for both the intervention and the comparison groups.

#### Types of outcome measures

To be included within this review, studies must have measured at least one of the following primary and/or secondary outcomes. We will look at maternal, fetal, neonatal and child health and nutrition outcomes that will help to inform related policy and practice. For simplifying, we have grouped all secondary outcomes of interest by these domains. Unless otherwise specified, all outcomes listed will be dichotomized (yes/no). We will use mean and standard deviation (SD) to report all continuous outcomes (maternal biochemical status, newborn anthropometry, newborn/child biochemical status). Outcome definitions can be found in brackets below. International Units (IU) will be used for all maternal outcomes whereas z‐scores will be used for child outcomes because of their adjustment for age.

Primary outcomes

► Maternal mortality (death while pregnant or within 42 days of pregnancy termination)
► Anemia/iron‐deficiency anaemia in third trimester of pregnancy [WHO 2011]
  ▷ Non‐anaemic: ≥110 g/L
  ▷ Anaemic: < 110 g/L
► Low birthweight (< 2500 g)
► Perinatal mortality (stillbirths and deaths ≤ 7 days)

Secondary outcomes

*Maternal outcomes:*

► Morbidity from trial enrolment up to three months post‐partum:
  ▷ Pre‐eclampsia/eclampsia
  ▷ Gestational hypertension
  ▷ Antepartum haemorrhage
  ▷ Postpartum haemorrhage
  ▷ Premature rupture of membranes
  ▷ Placental abruption
  ▷ Infections during pregnancy
  ▷ Bone mineral density
  ▷ Night blindness
  ▷ Need for blood transfusion
► Biochemical status at endline:
  ▷ Micronutrient deficiencies
    ■ Vitamin A (serum/plasma retinol) (continuous)
    ■ Iron (serum/plasma ferritin, plasma TfR, TIBC) (continuous)
    ■ Serum/plasma/red blood cell folate (continuous)
    ■ Serum/plasma zinc (continuous)
    ■ Serum/plasma alkaline phosphatase (continuous)
    ■ Serum/plasma copper (continuous)
    ■ Serum/plasma vitamin D (25‐hydroxyvitamin D) (continuous)
    ■ Thyroglobulin concentration (continuous)

*Fetal outcomes:*

► Mortality
  ▷ Miscarriage (loss of pregnancy before 28 weeks gestation)
  ▷ Stillbirth (death at or beyond 28 weeks' gestation)
► Morbidity
  ▷ Congenital anomalies

*Newborn outcomes:*

► Mortality:
  ▷ Neonatal mortality (deaths between 0 and 28 days)
► Morbidity:
  ▷ Preterm birth (< 37 weeks gestation)
  ▷ Small‐for‐gestational age (defined by study authors)
  ▷ Macrosomia (birthweight > 4000 g)
► Anthropometry measured from birth up to 14 days:
  ▷ Birth weight (z‐scores) (continuous)
  ▷ Birth length (z‐scores) (continuous)
  ▷ Head circumference (z‐scores) (continuous)

*Child outcomes:*

► Mortality
  ▷ Infant mortality (deaths between 0 and 12 months)
  ▷ Under‐five mortality (deaths between 0 and 59 months)
► Morbidity
  ▷ Stunting (‐2 z‐score or lower) at longest follow‐up
  ▷ Wasting (‐2 z‐score or lower) at longest follow‐up
  ▷ Underweight (‐2 z‐score or lower) at longest follow‐up
  ▷ Bone mineral density (continuous)
  ▷ Development outcomes (as defined by study authors)
  ▷ Infection
► Biochemical status at endline
  ▷ Micronutrient deficiencies
    ■ Vitamin A (serum/plasma retinol) (continuous)
    ■ Iron (serum/plasma ferritin, plasma TfR) (continuous)
    ■ Serum/plasma/red blood cell folate (continuous)
    ■ Serum/plasma zinc (continuous)
    ■ Serum/plasma vitamin D (25‐hydroxyvitamin D) (continuous)
► Anaemia
  ▷ Hemoglobin concentration (continuous)
► Iron deficiency anaemia

*Other outcomes:*

► Relevant long‐term outcomes during adolescence or adulthood, as specified by trial authors. For example:
  ▷ Anthropometrics (stunting, wasting, underweight) in children > 59 months
  ▷ Cognitive and motor development as assessed by trial investigators at longest follow‐up (e.g. Bayley Mental Development Index, Bayley Psychomotor Development Index, Stanford‐Binet test)
  ▷ Educational attainment (completion of primary or secondary school)
► Mode of delivery (vaginal, instrumental vaginal, caesarean)
► Adverse outcomes: any reported throughout intervention period (e.g. urinary tract infections, kidney stones, hyperthyroidism, allergic reactions, etc.), including short‐term adverse outcomes (e.g. vomiting, abdominal pain, constipation, diarrhoea, unpleasant tastes)

#### Duration of follow‐up

There will be no minimum duration of follow‐up.

#### Types of settings

Other than the LMIC inclusion criteria, there will be no restrictions regarding study setting.

Any *post hoc* changes to eligibility criteria or outcomes studied must be aligned with the review objectives and will be clearly stated with reasons justified.

### Search strategy

The search strategy will be guided by our PICO model (Table 1), but will not be restricted by outcome in order to retain a broader search. The search will be conducted using indexing terms, including medical subject headings (MeSH), keywords, and free text words. Details of the search strategy can be found in Appendix 2. To capture the most relevant evidence, we will include articles published from 1995 to the end of June 2018 (related programmes and good quality trials before 1995 were very limited). There will be no language or publication restrictions. Manual searches will be conducted within references lists of review articles and included studies, and experts will be contacted to obtain any additional relevant maternal that may have been missed. The search process, including month/year of search, will be documented to ensure that replication is possible.

**Table 1:** PICO table, used for formulating our search strategy

**Elements**	Description
P	Pregnant women of any age and parity, living in a low or middle‐income country
I	Micronutrient supplementation interventions ► Single and multiple micronutrient supplementation (including micronutrient powders) ► Lipid‐nutrient supplementation
C	Author‐defined
O	Primary: ► Anaemia/iron‐deficiency anaemia in pregnancy ► Low birthweight ► Perinatal mortality Secondary, maternal: ► Mortality ► Morbidity ► Micronutrient deficiencies Secondary, fetal: ► Mortality (miscarriage, stillbirth) ► Congenital anomalies Secondary, newborn: ► Mortality ► Morbidity (preterm, small‐for‐gestational‐age, macrosomia) ► Anthropometry (birth weight, birth length, head circumference) Secondary, child: ► Mortality ► Morbidity, including nutritional indicators (stunting, wasting, underweight) ► Micronutrient deficiencies ► Anaemia/iron‐deficiency anaemia Other secondary outcomes: ► Author‐specified long‐term outcomes in adolescence or adulthood ► Mode of delivery ► Adverse events

Electronic searches

The search will be run in the following databases, selected based on their applicability to the subject material:

► CAB Abstracts
► CINAHL
► Cochrane Central Register of Controlled Trials (CENTRAL)
► Embase
► International Initiative for Impact Evaluations (3ie)
► LILACS (Latin American and Caribbean health sciences literature)
► MEDLINE
► POPLINE
► Web of Science
► WHOLIS (WHO library database)

Searching other resources

*Unpublished studies*

► ProQuest Dissertations & Theses Global
► R4D (Research for Development) material from UK government's Department for International Development
► WHO International Clinical Trials Registry Platform (ICTRP)

*Grey literature*

Non‐indexed, grey literature searches will be conducted to locate relevant programme evaluations and any additional trials. We will search Google, Google Scholar, and web pages of key international nutrition agencies (listed below) using key words based on PICO methodology. We will use advanced search options, where possible. Google results will be screened online until no relevant result has appeared in three consecutive pages.

► Canadian Agency for Drugs and Technologies in Health (CADTH) tool for searching health‐related grey literature (http://www.cadth.ca/resources/finding‐evidence/grey‐matters)
► Centers for Disease Control and Prevention (CDC)
► Emergency Nutrition Network (ENN
► Global Alliance for Improved Nutrition (GAIN)
► Hellen Keller International
► International Food Policy and Research Institute (IFPRI)
► IZiNCG
► Nutrition International (NI)
► Sight and Life Foundation
► UNICEF
► World Food Programme (WFP)

### Data collection and analysis

#### Description of methods used in primary research

We anticipate that the vast majority of included studies will be randomised or cluster‐randomised controlled trials that follow our inclusion/exclusion criteria, as listed above. For example, in a study published by Christian and colleagues [Christian 2003], pregnant Nepalese women were cluster‐randomised to one of five groups: i) daily supplements of vitamin A (control), ii) daily supplements of vitamin A + folic acid, iii) daily supplements of vitamin A + folic acid + iron, iv) daily supplements of vitamin A + folic acid + iron + zinc, or v) daily multiple micronutrient supplements (including vitamin A) from early pregnancy to 72 hours postpartum. Outcomes assessed included infant anthropometrics (birth weight, length, and head circumference), and low birth weight (< 2500 grams).

#### Criteria for determination of independent findings

In order to take into account potential sources of dependency, we will group studies in terms of their location, population, the programme that is being evaluated (if applicable), and intervention type to ensure that there is no double counting of evidence when synthesizing results across studies. If there are multiple papers that describe the same trial, these will be combined and coded as a single study.

For trials that include multiple intervention arms, we will select one pair (intervention and control) that satisfy the inclusion criteria of the review and exclude the rest. If > 2 intervention groups meet the eligibility criteria, then these groups will be combined into a single pair‐wise comparison group and data will be disaggregated into corresponding subgroups, or these arms will be separated into different forest plots to ensure that there is no double counting of participants. We will analyze multiple outcome estimates within the same study separately.

#### Selection of studies

Two independent reviewers will perform title and abstract screening using specified inclusion/exclusion criteria. Where not enough information can be gleaned from the title alone, then abstracts will be screened in order to determine eligibility for full text screening. All full texts will then be screened in duplicate, with application of the same inclusion/exclusion criteria. A third reviewer will resolve any disagreements. Both title/abstract and full text screening will be done using Covidence, a web‐based software platform for systematic reviews. We will assess inter‐reviewer reliability/agreement by checking the number of conflicts in the Resolve Conflicts page following each stage of screening.

Examples of included studies:

► Mridha MK, Matias SL, Chaparro CM, et al. (2016) Lipid‐based nutrient supplements for pregnant women reduce newborn stunting in a cluster‐randomised controlled effectiveness trial in Bangladesh. Am J Clin Nutr; 103(1):236‐49.
► Roberfroid D, Huybregts L, Lamou H et al. (2008). Effects of maternal multiple micronutrient supplementation on fetal growth: a double‐blind, randomised controlled trial in rural Burkina Faso. Am J Clin Nutr; 88:1330‐40.

Examples of excluded studies:

► Harvey LJ, Dainty JR, Hollands WJ, et al. (2007) Effect of high‐dose iron supplements on fractional zinc absorption and status in pregnant women. Am J of Clin Nutr; 85:131‐6.
  ▷ Ineligible population (high‐income setting)
► Boran P, Tokuc G, Vagas E, et al. (2006) Impact of zinc supplementation in children with acute diarrhoea in Turkey. Arch Dis Child; 91(4):296‐99.
  ▷ Ineligible population (children six months to five years of age)

#### Data extraction and management

For all included studies, we will extract data into a standardized data abstraction form that is comprised of a general study information sheet and a quantitative outcomes sheet. The data abstraction form will be piloted before it is finalized. While all arms of a study will be described in the tables of included studies, data will be extracted and reported on only for those arms that meet review criteria. All data abstraction will be performed in duplicate. Coders will be trained in systematic review methods, and data abstraction will be cross‐checked with primary study data for accuracy by the team lead.

Each general study information sheet will contain the following:

► General study information: authors, publication year, language of study, study design
► Study setting: World Bank region, country, World Bank income level, city/town, urban/urban slum/rural/mixed setting, duration of data collection, date of data collection
► Study population: sample size recruited, sample size analysed, male/female/mixed (%), age range of participants, mean/median age of participants, description of participants (i.e. inclusion/exclusion criteria applied to recruitment)
► Intervention characteristics: type of intervention, food vehicle utilized (where applicable), duration of intervention, level of delivery, unit of randomisation (where applicable), dose of micronutrient(s) provided, frequency of provision (i.e. daily, weekly, etc.), duration of follow‐up, attrition rate
► Programmatic indicators (based on the WHO/CDC logic model [De‐Regil 2014]): policies, production, delivery strategies, quality control, behaviour change communication strategies, access and coverage, knowledge and appropriate use
► Funding source of programme (where applicable)
► Quality assessment (see section below: critical appraisal of studies

Each quantitative outcome sheet will contain the following:

► Subgroup (if applicable)
► Subgroup sample size
► Outcome type (based on outcomes listed above)
► Outcome units
► Outcomes:
  ▷ Outcome measure treatment group
  ▷ Outcome measure comparison group
  ▷ Standard deviation
► Effect size:
  ▷ Effect measure (specify type); unadjusted and adjusted
  ▷ 95% confidence interval
  ▷ P value of effect measure
  ▷ Standard error (SE) or standard deviation (SD) or t‐statistic

#### Assessment of risk of bias in included studies

We will critically appraise individual studies using the Cochrane Effective Practice and Organisation of Care (EPOC) guidelines for randomised trials, non‐randomised trials, controlled before‐after studies, and interrupted time series (ITS) studies. EPOC guidelines include the following standardized criteria for assessing bias of randomised, non‐randomised, and controlled before‐after studies [EPOC 2017]:

► Random sequence generation
► Allocation concealment
► Baseline outcome measurements similar
► Baseline characteristics similar
► Incomplete outcome data
► Knowledge of the allocated interventions adequately prevented during study
► Protection against contamination
► Selective outcome reporting
► Other risks of bias (e.g. bias in measurement: validity and reliability of the measures used)

For ITS studies, the following criteria will be considered [EPOC 2017]:

► Intervention independent of other changes
► Shape of intervention effect pre‐specified
► Intervention unlikely to affect data collection
► Knowledge of the allocated interventions adequately prevented during study
► Incomplete outcome data
► Selective outcome reporting
► Other risks of bias (e.g. bias in measurement: validity and reliability of the measures used)

For EPOC rating schemes for randomised trials, non‐randomised trials, and controlled before‐after studies, please see Table 2 and for interrupted time series studies, see Table 3.

**Table 2:** EPOC criteria for assessing risk of bias in randomised trials, non‐randomised trials, and controlled before‐after studies

**Criteria**	Rating Scheme
Random sequence generation	Low risk if a random component in the sequence generation process is described
	High risk when a non‐random method is used
	Unclear risk if not specified in the paper
Allocation concealment	Low risk if the unit of allocation was by institution, team or professional and allocation was performed on all units at the start of the study; or if the unit of allocation was by patient or episode of care and there was some form of centralised randomizations scheme, an on‐site computer system or sealed opaque envelopes were used
	Controlled before‐after studies are scored high risk
	Unclear risk if not specified in the paper
Baseline outcome measurements similar	Low risk if performance or patient outcomes were measured prior to the intervention, and no important differences were present across study groups. In randomised trials, score low irks if imbalanced bur appropriate adjusted analysis was performed
	High risk if important differences were present and not adjusted for in analysis
	If randomised trials have no baseline measure of outcome, score unclear risk
Baseline characteristics similar	Low risk if baseline characteristics of the study and control providers are reported and similar
	High risk if there is no report of characteristics in text or tables or if there are differences between control and intervention providers
	Unclear risk if it is not clear in the paper (e.g. characteristics are mentioned in text but no data were provided)
Incomplete outcome data	Low risk if missing outcome measures were unlikely to bias the results (e.g. the proportion of missing data were similar in the intervention and control groups or the proportion of missing data were less than the effect size)
	High risk if missing outcome data were likely to bias the results
	Unclear risk of not specified in the paper (without assuming 100% follow‐up unless explicitly stated)
Knowledge of the allocated interventions adequately prevented during study	Low risk if authors state explicitly that the primary outcome variables were assessed blindly, or the outcomes are objective
	High risk if the outcomes were not assessed blindly
	Unclear risk if not specified in the paper
Protection against contamination	Low risk if allocation was by community, institution or practice and it is unlikely that the control group received the intervention
	High risk if it is likely that the control group received the intervention
	Unclear risk if professionals were allocated within a clinic or practice and it is possible that communication between intervention and control professionals could have occurred
Selective outcome reporting	Low risk if there is no evidence that outcomes were selectively reported
	High risk if some important outcomes are omitted from the results
	Unclear risk if not specified in the paper
Other risks of bias (e.g. bias in measurement: validity and reliability of the measures used)	Low risk if there is no evidence of other risk of biases

**Table 3:** EPOC criteria for assessing risk of bias in interrupted time series studies

**Criteria**	Rating scheme
Intervention independent of other changes	Low risk if there are compelling arguments that the intervention occurred independently of other changes over time and the outcome was not influenced by other confounding variables/historic events during study period
	High risk if reported that intervention was not independent of other changes in time
	Unclear risk if not specified in the paper
Shape of intervention effect pre‐specified	Low risk if point of analysis is the point of intervention or a rational explanation for the shape of intervention effect was given by the author(s)
	High risk if it is clear that the condition above is not met
	Unclear risk if not specified in the paper
Intervention unlikely to affect data collection	Low risk if reported that intervention itself was unlikely to affect data collection (for example, sources and methods of data collection were the same before and after the intervention)
	High risk if the intervention itself was likely to affect data collection (for example, any change in source or method of data collection reported)
	Unclear risk if not specified in the paper
Knowledge of the allocated interventions adequately prevented during study	Low risk if the authors state explicitly that the primary outcome variables were assessed blindly, or the outcomes are objective, e.g. length of hospital stay
	High risk if the outcomes were not assessed blindly
	Unclear risk if not specified in the paper
Incomplete outcome data	Low risk if missing outcome measures were unlikely to bias the results (e.g. the proportion of missing data were similar in the pre‐ and post‐intervention periods or the proportion of missing data were less than the effect size i.e. unlikely to overturn the study result)
	High risk if missing outcome data were likely to bias the results
	Unclear risk if not specified in the paper (Do not assume 100% follow‐up unless stated explicitly)
Selective outcome reporting	Low risk if there is no evidence that outcomes were selectively reported (e.g. all relevant outcomes in the methods section are reported in the results section)
	High risk if some important outcomes are subsequently omitted from the results
	Unclear risk if not specified in the paper
Other risks of bias (e.g. bias in measurement: validity and reliability of the measures used)	Low risk if there is no evidence of other risk of biases

In addition, the Cochrane risk of bias (ROB) tool [Higgins 2011] will be used for randomised studies, including cluster‐randomised trials and step‐wedge designs. The ROB tool uses the following criteria for assessment of bias. Of note, we will assess performance and detection bias separately.

► Selection bias: random sequence generation and allocation concealment
► Performance bias: blinding of participants and personnel
► Detection bias: blinding of outcome assessment
► Attrition bias: incomplete outcome data
► Reporting bias: selective reporting
► Other sources of bias

All risk of bias assessments will be performed in duplicate and supportive evidence for all ROB judgements will be documented. A third reviewer will resolve any disagreements. An overall score will not be provided.

#### Measures of treatment effect

We will convert data for each outcome into the same format (e.g. means and standard deviations for continuous data), including appropriate conversion of scales such that an increase/decrease always indicates improvement or deterioration of an indicator. If included studies have data that are reported in a not ‘usable’ way (i.e., data cannot be pooled with other data), we will retain the study as eligible but will restrict it from further analysis.

We will analyze dichotomous and continuous outcomes separately. For dichotomous outcomes, results will be presented as summary risk ratios (RRs) with 95% CIs, whenever possible, in order to compare risk of the outcome between intervention and control groups. When including incidence data, we will combine risk ratios (events per child) and rate ratios (events per child year) because of their similar interpretation and scale. We will present continuous outcome data as either a mean difference (MD), if outcomes have been measured on the same scale, or a standardized mean difference (SMD), if outcomes have been measured on different scales, with 95% CIs. Both change from baseline scores and final measurements (for RCTs only) will be eligible, and can be pooled where there is meta‐analysis with MD (i.e., scales are the same and measurements are in the same unit) [Higgins 2011]. We will carefully consider reporting of the appropriate means and standard deviations (either of final measurements or of changes from baseline) if both change and final values are used in one meta‐analysis. We will not combine final values and changes scores as SMDs because the standard deviation in this case reflects differences in measurement reliability. Where it is necessary to combine measures of treatment effect with SMDs, we will use change scores, given that the standard deviation of the change is also reported.

#### Unit of analysis issues

All interventions and, within those interventions, outcomes will be meta‐analysed separately. We will also meta‐analyse RCTs and quasi‐experimental studies separately.

Special attention will be given to cluster‐randomised trials; this is to ensure that clustering has been appropriately accounted for within the analysis of the primary study, such that study precision is not over or under‐estimated within our analysis. If necessary, we will adjust effect estimates of cluster‐randomised trials using the mean cluster size (M) and the intra‐cluster correlation coefficient (ICC), which quantifies the extent to which data from the same cluster are correlated [design effect = 1 + (M‐1) ICC]. The design effect will then be used to adjust the study data such that a trial is reduced to its effective sample size. We will not make any adjustments if authors have appropriately adjusted for cluster design already.

Randomized and non‐randomised studies with contemporaneous comparison groups will be analysed separately, but may be pooled if differences in findings are not statistically significant. We will analyze and report findings from controlled before‐after and ITS study designs separately.

#### Dealing with missing data

Where data are incomplete or in a form that cannot be converted with the information available, we will contact the corresponding author for clarification or to obtain missing data. If authors have accounted for missing data (i.e. multiple imputations), we will use the adjusted data within our analysis.

#### Assessment of heterogeneity

Statistical heterogeneity will be assessed using Tau^2^, I^2^ and significance of the Chi‐square test; we will also assess heterogeneity visually using forest plots. Based on prior theory and clinical knowledge, we expect clinical and methodological heterogeneity in effect sizes in this literature. Therefore, we will attempt to explain any observed statistical heterogeneity using subgroup analysis (see below).

#### Assessment of reporting biases

If the number of studies is sufficient (> 10), funnel plots will be used to visually assess publication bias. This kind of bias is unlikely if data forms a symmetric inverted funnel shape around the mean effect estimate. In addition, we will perform Egger's test to determine funnel plot asymmetry.

#### Data synthesis

Statistical analysis will be carried out using Review Manager 5.3 and Stata. We will follow intention to treat analysis for RCTs. We will reconstruct the data to create an intention to treat analysis where authors have reported a per protocol analysis.

Random‐effects meta‐analysis will be used to account for any expected heterogeneity in interventions, comparisons, outcomes, or settings within the studies included in a given synthesis. Where meta‐analysis is deemed inappropriate due to substantial methodological or statistical heterogeneity between studies, we will summarize the findings of the included studies in narrative or table form.

The generic inverse‐variance approach will be used for both dichotomous and continuous outcomes, such that the study weights will be adjusted according to the variance of the effect estimate (i.e. the larger studies with smaller standard error will be given more weight than smaller studies with larger standard error). For random‐effects analyses, the DerSimonian and Laird method will be applied to incorporate a measure of variation (Tau^2^) among intervention effects from different studies.

We will use raw summary estimates to construct meta‐analyses from RCTs and adjusted estimates to construct meta‐analyses from observational studies. We will interpret overall effect estimates that have an associated p‐value < 0.05 as statistically significant. We will also report non‐significant findings. Where possible, interaction tests will be used to determine if there is a relevant difference in effect across subgroups. We will base the conclusion that an intervention is effective in one subgroup but not another on a direct test of the mean difference between two groups (i.e., with meta‐regression).

We will use the GRADE tool to assess the body of evidence for selected outcomes for which a meta‐analysis has been conducted. We have chosen the following outcomes: maternal mortality, maternal anemia, low birthweight, and perinatal mortality. We will summarize this assessment in a ‘Summary of Findings’ table, created with GRADE pro software. We will rate the quality of the body of evidence for each selected outcome as ‘high’, ‘moderate’, ‘low’ or ‘very low’. Randomized trials will initially be rated as high quality evidence, but they may be downgraded according to the five criteria listed below. Quasi‐experimental studies will initially be rated as low quality evidence, but they can be upgraded if they do not have any serious methodological limitations. They can also be downgraded further.

There are five criteria that can downgrade evidence [GRADE 2004]:

► Risk of bias in individual studies
► Indirectness of evidence
► Unexplained heterogeneity or inconsistency of results
► Imprecision of results
► High probably of publication bias

There are three criteria that can upgrade the evidence for quasi‐experimental studies with no serious methodological limitations. [GRADE 2004]:

► Large magnitude of effect
► Presence of a dose response relationships
► Effect of plausible residual confounding

Quality ratings, as determined by GRADE, are found in Table 4.

**Table 4:** Quality of evidence, as determined by GRADE criteria

**Study**	Outcome
Very low	Any estimate of effect is uncertain.
Low	Further research is very likely to have an important impact on our confidence in the estimate of effect and is likely to change the estimate.
Moderate	Further research is likely to have an important impact on our confidence in the estimate of effect and may change the estimate.
High	Further research is very unlikely to change our confidence in the estimate of effect.

#### Subgroup analysis and investigation of heterogeneity

Heterogeneity will be assessed based on clinical knowledge and theory, and investigation of statistical criteria such as Tau^2^, I^2^ and significance of the Chi‐sqaure test.

Depending on data availability (≥3 studies per subgroup of interest), we will conduct subgroup analyses on the primary outcomes for the following variables:

► Age (10‐14 years, 15‐19 years, 20‐29 years, 30‐39 years, 40+)
► Geographical region (based on WHO regions)
► Sex of infant
► Baseline nutritional status
  ▷ Anaemic versus non‐anaemic
  ▷ Undernutrition versus normal nutrition, based on BMI (BMI < 18.5)
  ▷ Low stature versus normal stature
► Duration of intervention
  ▷ Women recruited prior to conception versus first trimester versus second trimester versus third trimester of pregnancy
► Frequency of intervention
  ▷ Daily versus intermittent IFA supplementation
► Dose of intervention
  ▷ 30 mg versus 60 mg elemental iron for IFA, MMN, or LNS supplementation
► UNIMMAP versus adapted UNIMMAP versus non‐UNIMMAP formulations for MMN supplementation [UNICEF, WHO, and UNU 1999]
  ▷ MMN supplements that contain a similar number and type of vitamins and minerals as the UNIMMAP formulation will be categorized as ‘adapted UNIMMAP’ (± 2 micronutrients, when compared to the standard UNIMMAP formulation)
  ▷ Supplements with the same composition as UNIMMAP but different doses of vitamins and minerals can also be categorized as ‘adapted UNIMMAP’

Variables were selected a priori, based on evidence to support their potential to impact the intervention effect. We will carefully interpret results from subgroup analyses. We will also use meta‐regression techniques to assess how characteristics of studies (explanatory variables) may influence the size of the effect estimate (outcome variable). Potential variables may include the setting, dosing frequency, dosing form, compound, duration, route, sex of infant, SES status, or nutritional status. Any subgroup analysis that is conducted *post hoc* will be exploratory in nature and will be stated as such.

#### Sensitivity analysis

Sensitivity analyses will be conducted to determine whether the removal of studies with high risk of bias or the removal of non‐randomised studies significantly impacts findings. We will define studies as having a high risk of bias if one or more domains have been judged as ‘high risk’ or two or more domains have been judged as ‘unclear risk’.

#### Treatment of qualitative research

We do not plan to include qualitative research.

## Review authors

**Lead review author:** The lead author is the person who develops and co‐ordinates the review team, discusses and assigns roles for individual members of the review team, liaises with the editorial base and takes responsibility for the on‐going updates of the review.

**Table:** 

Name:	Emily C. Keats
Title:	Research Associate
Affiliation:	Centre for Global Child Health
Address:	686 Bay Street suite 11.9805
City, State, Province or County:	Toronto, ON
Post code:	M5G 0A4
Country:	Canada
Phone:	+1 416 813 7654 x 309518
Email:	Emily.keats@sickkids.ca
**Co‐authors:**	
Name:	Aamer Imdad
Title:	Assistant Profressor of Pediatrics
Affiliation:	State University of New York Upstate Medical University
Address:	Suite 504, 725 Irving Ave
City, State, Province or County:	Syracuse, NY
Post code:	13210
Country:	USA
Phone:	+1 315 447 5407
Email:	Aamer08@gmail.com
	
Name:	Zulfiqar A. Bhutta
Title:	Co‐Director
Affiliation:	Centre for Global Child Health
Address:	686 Bay Street suite 11.9805
City, State, Province or County:	Toronto, ON
Post code:	M5G 0A4
Country:	Canada
Phone:	+1 416 813 7654 x 301774
Email:	Zulfiqar.bhutta@sickkids.ca

## Roles and responsibilities

Emily Keats and Aamer Imdad have methodological, statistical, and information retrieval expertise. Zulfiqar Bhutta has content expertise. All additional team members will receive training in systematic review methods.

## Sources of support

Funding for this review came from a grant from the Bill & Melinda Gates Foundation to the Centre for Global Child Health at The Hospital for Sick Children (Grant No. OPP1137750).

## Declarations of interest

The authors are not aware of any conflicts of interest arising from financial or researcher interests.

## Preliminary timeframe

Approximate date for submission of the systematic review: February, 2019

## Plans for updating the review

The corresponding author, Dr. Zulfiqar A. Bhutta, will be responsible for any forthcoming updates to the review.

## AUTHOR DECLARATION

### Authors' responsibilities

By completing this form, you accept responsibility for preparing, maintaining and updating the review in accordance with Campbell Collaboration policy. Campbell will provide as much support as possible to assist with the preparation of the review.

A draft review must be submitted to the relevant Coordinating Group within two years of protocol publication. If drafts are not submitted before the agreed deadlines, or if we are unable to contact you for an extended period, the relevant Coordinating Group has the right to de‐register the title or transfer the title to alternative authors. The Coordinating Group also has the right to de‐register or transfer the title if it does not meet the standards of the Coordinating Group and/or Campbell.

You accept responsibility for maintaining the review in light of new evidence, comments and criticisms, and other developments, and updating the review at least once every five years, or, if requested, transferring responsibility for maintaining the review to others as agreed with the Coordinating Group.

### Publication in the Campbell Library

The support of the Coordinating Group in preparing your review is conditional upon your agreement to publish the protocol, finished review, and subsequent updates in the Campbell Library. Campbell places no restrictions on publication of the findings of a Campbell systematic review in a more abbreviated form as a journal article either before or after the publication of the monograph version in Campbell Systematic Reviews. Some journals, however, have restrictions that preclude publication of findings that have been, or will be, reported elsewhere and authors considering publication in such a journal should be aware of possible conflict with publication of the monograph version in Campbell Systematic Reviews. Publication in a journal after publication or in press status in Campbell Systematic Reviews should acknowledge the Campbell version and include a citation to it. Note that systematic reviews published in Campbell Systematic Reviews and co‐registered with Cochrane may have additional requirements or restrictions for co‐publication. Review authors accept responsibility for meeting any co‐publication requirements.

**I understand the commitment required to undertake a Campbell review, and agree to publish in the Campbell Library. Signed on behalf of the authors**:

**Table:** 

**Form completed by: Emily Keats**	**Date: 16 November 2018**

## Appendix 1: Existing reviews

### Iron folic acid supplementation:

Peña‐Rosas JP, De‐Regil LM, Gomez Malave H, Flores‐Urrutia MC, Dowswell T. Intermittent oral iron supplementation during pregnancy. Cochrane Database Syst Rev 2015 Oct 19;(10):CD009997. doi:10.1002/14651858.CD009997.pub2.

Peña‐Rosas JP, De‐Regil LM, Garcia‐Casal MN, Dowswell T. Daily oral iron supplementation during pregnancy. Cochrane Database Syst Rev 2015, Issue 7. Art. No.: CD004736. doi:10.1002/14651858.CD004736.pub5.

Haider BA, Olofin I, Wang M, Spiegelman D, Ezzati M, Fawzi WW; Nutrition Impact Model Study Group (anaemia). Anaemia, prenatal iron use, and risk of adverse pregnancy outcomes: systematic review and meta‐analysis. BMJ 2013;21;346:f3443. doi:10.1136/bmj.f3443.

### Multiple micronutrient supplementation:

Haider BA, Bhutta ZA. Multiple‐micronutrient supplementation for women during pregnancy. Cochrane Database Syst Rev. 2017 Apr 13;4:CD004905. doi: 10.1002/14651858.CD004905.pub5.

Suchdev PS, Peña‐Rosas JP, De‐Regil LM. Multiple micronutrient powders for home (point‐of‐use) fortification of foods in pregnant women. Cochrane Database Syst Rev 2015, Issue 6. Art. No: CD011158. doi:10.1002/14651858.CD011158.pub2.

### Single micronutrient supplementation:

Lassi ZS, Salam RA, Haider BA, Bhutta ZA. Folic acid supplementation during pregnancy for maternal health and pregnancy outcomes. Cochrane Database Syst Rev 2013 Mar 28;(3):CD006896. doi:10.1002/14651858.CD006896.pub2.

Hodgetts VA, Morris RK, Francis A, Gardosi J, Ismail KM. Effectiveness of folic acid supplementation in pregnancy on reducing the risk of small‐for‐gestational age neonates: a population study, systematic review and meta‐analysis. BJOG 2015;122:478–90.

Buppasiri P, Lumbiganon P, Thinkhamrop J, Ngamjarus C, Laopaiboon M, Medley N. Calcium supplementation (other than for preventing or treating hypertension) for improving pregnancy and infant outcomes. Cochrane Database Syst Rev 2015, Issue 2. Art. No.: CD007079. doi:10.1002/14651858.CD007079.pub3.

Hofmeyr GJ, Lawrie TA, Atallah ÁN, Duley L, Torloni MR. Calcium supplementation during pregnancy for preventing hypertensive disorders and related problems. Cochrane Database Syst Rev 2014, Issue 6. Art. No.: CD001059. doi:10.1002/14651858.CD001059.pub4.

Harding KB, Peña‐Rosas JP, Webster AC, Yap CMY, Payne BA, Ota E, De‐Regil LM. Iodine supplementation for women during the preconception, pregnancy and postpartum period. Cochrane Database Syst Rev 2017, Issue 3. Art. No.: CD011761. doi:10.1002/14651858.CD011761.pub2

De‐Regil LM, Palacios C, Lombardo LK, Peña‐Rosas JP. Vitamin D supplementation for women during pregnancy. Cochrane Database Syst Rev 2016, Issue 1. Art. No: CD008873. doi:10.1002/14651858.CD008873.pub3.

Ota E, Mori R, Middleton P, Tobe‐Gai R, Mahomed K, Miyazaki C, Bhutta ZA. Zinc supplementation for improving pregnancy and infant outcomes. Cochrane Database Syst Rev 2015, Issue 2. Art. No.: CD000230. doi: 10.1002/14651858.CD000230.pub5.

### Lipid‐based nutrient supplementation:

Das JK, Salam RA, Weise PZ, et al. Lipid‐based nutrient supplements for pregnant women and their impact on pregnancy, birth, and infant developmental outcomes in stable and emergency settings. 2017 Cochrane (review protocol).

## Appendix 2: Search strategy

Population:

1. Pregnant women/ or Mothers/ or Maternal Age/ or Pregnancy in Adolescence/ or Pregnancy, Multiple/ or exp Perinatology/ or First Trimester, Pregnancy/ or Second Trimester, Pregnancy/ or Third Trimester, Pregnancy/ or Obstetrics/ or exp Maternal Behavior/ or Surrogate Mothers/ or Pregnancy Outcome/ or Prenatal Care/ or Perinatal Care/ or Postnatal Care/ or Postpartum Period/ or Lactation/ or Pregnancy Complications/ or (pregnan* or mother* or mom* or surroga* or matern* or preconcept* or pre‐concept* or “pre concept*” or periconcept* or peri‐concept* or “peri concept*” or partus or lactation or obstetric* or labo#r or childbear* or “child‐bear*” or gestation* or antenatal or ante‐natal or “ante natal” or pre‐natal or “pre natal” or prenatal or perinatal or peri‐natal or “peri natal” or prepartum or pre‐partum or “pre partum” or perinatology or peripartum or peri‐partum or “peri partum” or puerper* or postpartum or post‐partum or “post partum” or postnatal or post‐natal or “post natal” or “child birth” or child‐birth or childbirth or “term birth” or paturiency or child‐carrying or “child carrying” or (pregnan* and (“reproductive age” or “wom#n of reproductive age” or WRA))).tw,kf

Single or Multiple Micronutrient Supplementation:

2. Micronutrients/ or vitamins/ or minerals/ or exp iron/ or exp iron compounds/ or iron, dietary/ or vitamin A/ or exp iodine/ or exp zinc or exp zinc compounds/ or exp vitamin D/ or (micronutrient* or multinutrient* or multi‐nutrient* or “multi*nutrient” or “multimicro‐nutrient*” or “multimicronutrient*” or multivitamin* or “multi‐vitamin*” or multimineral* or “multi‐mineral*” or “multiple micro nutrient*” or “multiple micronutrient” or micro‐nutrient* or MMN or “essential vitamins*” or mineral* or “m.v.i. pediatric” or “trace element*” or “trace mineral*” or “trace metal” or vitamin* or “vitamin d” or “hydroxyvitamin d” or vitamin‐d or “25 hydroxyvitamin d” or “25 hydroxy‐vitamin d” or “25‐hydroxyvitamin d” or “25‐hydroxy‐vitamin d” or “25‐hydroxyvitamin d” or 25ohd or “25‐oh‐vitamin d” or 25‐ohd or “vitamin d2” or vitamin‐d2 or “25‐hydroxyvitamin d2” or “25‐hydroxy‐vitamin d2” or “vitamin d3” or vitamin‐d3 or “25 hydroxyvitamin d3” or “25 hydroxyvitamin d3” or “25‐hydroxy‐vitamin d3” or calcidiol or calcifediol or calcium or retinol* or retinal* or Retinaldehyde or retinoid or Retinoids or retinoic or beta‐carotene or “beta carotene” or iron or “Fe(III)” or “Fe3+” or “iron(III)” or “Ferrous ion” or “Fe(II)” or “iron(II)” or “Fe2+” or “ferr* compounds” or zinc or “zn” or “zn acetate” or “zn sulfate” or “zn oxide” or iodine or “iod* compounds” or “ferr* compounds” or “folic acid” or “ergocalciferol derivative” or “ergocalciferol‐D2” or cholecalciferol‐D3 or “colecalciferol derivative” or iodiz* or “beta carotene” or “b‐tene” or “beta carotin” or betacarotene).tw,kf

3. Exp Dietary supplements/ or tablets/ or syrup/ or capsules/ or powders/ or (supplement* or nutraceutical* or nutriceutical* or neutraceutical* or capsule* or tablet* or syrup* or drop* or Sprinkles or powder* or foodlet* or “foodlet‐based” or “crushable nutritabs” or “micronutrient powder*” or “multiple‐micronutrient powder” or mnp).tw,kf

4. 1 AND 2 AND 3

Lipid‐Nutrient Supplementation:

5. exp Lipids/ or (lipid* or oil* or soy* or peanut* or whey* or sesame* or cashew* or chickpea* or protein* or butter* or fat or fats or fatty or “dairy product*” or “omega‐3” or “omega 3” or “alpha‐linolenic acid” or “docosahexaenoic acids” or “eicosapentaeonic acid” or “n‐3 pufa” or “n3 pufa” or glyceride*).tw,kf

6. Dietary supplements/ or (“lipid based” or “lipid‐based nutri*” or enrich* or emuls* or “Lipid Emulsions” or “Fat Emulsions” or “Intravenous Fat Emulsions” or powder* or spread* or paste* or LNS or iLiNS or supplement* or neutraceutical* or nutraceutical* or nutriceutical* or Nutributter* or Plumpy* or PlumpyNut or “ready to use” or “ready‐to‐use therapeutic food” or “ready‐to‐use supplementary food” or RUFF or RUSF or RUTF).tw,kf

7. 1 AND 5 AND 6

Low or Middle‐Income Country:

8. Developing Countries/ or developing country*.tw, kf or Afghanistan/ or Guinea/ or Rwanda/ or Benin/ or Guinea‐Bissau/ or Senegal/ or “Burkina Faso”/ or Haiti/ or “Sierra Leone”/ or Burundi/ or “Democratic People's Republic of Korea”/ or Somalia/ or “Central African Republic”/ or Liberia/ or “South Sudan”/ or Chad/ or Madagascar/ or Tanzania/ or Comoros/ or Malawi/ or Togo/ or “Democratic Republic of the Congo”/ or Mali/ or Uganda/ or Eritrea/ or Mozambique/ or Zimbabwe/ or Ethiopia/ or Nepal/ or “The Gambia”/ or Niger/ or Angola/ or Indonesia/ or Philippines/ or Armenia/ or Jordan/ or “São Tomé and Principe”/ or Bangladesh/ or Kenya/ or “Solomon Islands”/ or Bhutan/ or Kiribati/ or “Sri Lanka”/ or Bolivia/ or Kosovo/ or Sudan/ or “Cabo Verde”/ or “Kyrgyz Republic”/ or Swaziland/ or Cambodia/ or “Lao PDR”/ or “Syrian Arab Republic”/ or Cameroon/ or Lesotho/ or Tajikistan/ or “Republic of the Congo”/ or Mauritania/ or Timor‐Leste/ or “Côte d'Ivoire”/ or “Federated States of Micronesia”/ or Tunisia/ or Djibouti/ or Moldova/ or Ukraine/ or “Arab Republic of Egypt”/ or Mongolia/ or Uzbekistan/ or “El Salvador”/ or Morocco/ or Vanuatu/ or Georgia/ or Myanmar/ or Vietnam/ or Ghana/ or Nicaragua/ or “West Bank and Gaza”/ or Guatemala/ or Nigeria/ or “Republic of Yemen”/ or Honduras/ or Pakistan/ or Zambia/ or India/ or “Papua New Guinea”/ or Albania/ or Ecuador/ or Nauru/ or Algeria/ or Fiji/ or Panama/ or “American Samoa”/ or Gabon/ or Paraguay/ or Argentina/ or Grenada/ or Peru/ or Azerbaijan/ or Guyana/ or Romania/ or Belarus/ or “Islamic Republic of Iran”/ or “Russian Federation”/ or Belize/ or Iraq/ or Samoa/ or “Bosnia and Herzegovina”/ or Jamaica/ or Serbia/ or Botswana/ or Kazakhstan/ or “South Africa”/ or Brazil/ or Lebanon/ or “St. Lucia”/ or Bulgaria/ or Libya/ or “St. Vincent and the Grenadines”/ or China/ or “Republic of Macedonia”/ or Suriname/ or Colombia/ or Malaysia/ or Thailand/ or “Costa Rica”/ or Maldives/ or Tonga/ or Croatia/ or “Marshall Islands”/ or Turkey/ or Cuba/ or Mauritius/ or Turkmenistan/ or Dominica/ or Mexico/ or Tuvalu/ or “Dominican Republic”/ or Montenegro/ or “Bolivarian Republic of Venezuela”/ or “Equatorial Guinea”/ or Namibia/ or (“developing country” or “developing countries” or “developing nation” or “developing nations” or “developing population” or “developing populations” or “developing world” or “less developed country” or “less developed countries” or “less developed nation” or “less developed nations” or “less developed population” or “less developed populations” or “less developed world” or “lesser developed country” or “lesser developed countries” or “lesser developed nation” or “lesser developed nations” or “lesser developed population” or “lesser developed populations” or “lesser developed world” or “under developed country” or “under developed countries” or “under developed nation” or “under developed nations” or “under developed population” or “under developed populations” or “under developed world” or “underdeveloped country” or “underdeveloped countries” or “underdeveloped nation” or “underdeveloped nations” or “underdeveloped population” or “underdeveloped populations” or “underdeveloped world” or “middle income country” or “middle income countries” or “middle income nation” or “middle income nations” or “middle income population” or “middle income populations” or “low income country” or “low income countries” or “low income nation” or “low income nations” or “low income population” or “low income populations” or “lower income country” or “lower income countries” or “lower income nation” or “lower income nations” or “lower income population” or “lower income populations” or “underserved country” or “underserved countries” or “underserved nation” or “underserved nations” or “underserved population” or “underserved populations” or “underserved world” or “under served country” or “under served countries” or “under served nation” or “under served nations” or “under served population” or “under served populations” or “under served world” or “deprived country” or “deprived countries” or “deprived nation” or “deprived nations” or “deprived population” or “deprived populations” or “deprived world” or “poor country” or “poor countries” or “poor nation” or “poor nations” or “poor population” or “poor populations” or “poor world” or “poorer country” or “poorer countries” or “poorer nation” or “poorer nations” or “poorer population” or “poorer populations” or “poorer world” or “developing economy” or “developing economies” or “less developed economy” or “less developed economies” or “lesser developed economy” or “lesser developed economies” or “under developed economy” or “under developed economies” or “underdeveloped economy” or “underdeveloped economies” or “middle income economy” or “middle income economies” or “low income economy” or “low income economies” or “lower income economy” or “lower income economies” or “low gdp” or “low gnp” or “low gross domestic” or “low gross national” or “lower gdp” or “lower gnp” or “lower gross domestic” or “lower gross national” or lmic or lmics or “third world” or “lami country” or “lami countries” or “transitional country” or “transitional countries” or Africa or Asia or Caribbean Region or West Indies or South America or Latin America or Central America or Afghanistan or Albania or Algeria or Angola or Argentina or Armenia or Armenian or Azerbaijan or Bangladesh or Benin or Byelarus or Byelorussian or Belarus or Belorussian or Belorussia or Belize or Bhutan or Bolivia or Bosnia or Herzegovina or Hercegovina or Botswana or Brazil or Bulgaria or Burkina Faso or Burkina Fasso or Upper Volta or Burundi or Urundi or Cambodia or Khmer Republic or Kampuchea or Cameroon or CameroonsOR Cameron or Camerons or Cape Verde or Central African Republic or Chad or China or Colombia or Comoros or Comoro Islands or Comores or Mayotte or Congo or Zaire or Costa Rica or Cote d'Ivoire or Ivory Coast or Cuba or Djibouti or French Somaliland or Dominica or Dominican Republic or East Timor or East Timur or Timor Leste or Ecuador or Egypt or United Arab Republic or El Salvador or Eritrea or Ethiopia or Fiji or Gabon or Gabonese Republic or Gambia or Gaza or Georgia Republic or Georgian Republic or Ghana or Gold Coast or Grenada or Guatemala or Guinea or Guiana or Guyana or Haiti or Honduras or India or Maldives or Indonesia or Iran or Iraq or Isle of Man or Jamaica or Jordan or Kazakhstan or Kazakh or Kenya or Kiribati or Korea or Kosovo or Kyrgyzstan or Kirghizia or Kyrgyz Republic or Kirghiz or Kirgizstan or “Lao PDR” or Laos or Lebanon or Lesotho or Basutoland or Liberia or Libya or Macedonia or Madagascar or Malagasy Republic or Malaysia or Malaya or Malay or Sabah or Sarawak or Malawi or Nyasaland or Mali or Marshall Islands or Mauritania or Mauritius or Agalega Islands or Mexico or Micronesia or Middle East or Moldova or Moldovia or Moldovian or Mongolia or Montenegro or Morocco or Ifni or Mozambique or Myanmar or Myanma or Burma or Namibia or Nepal or Nicaragua or Niger or Nigeria or Muscat or Pakistan or Palau or Palestine or Panama or Paraguay or Peru or Philippines or Philipines or Phillipines or Phillippines or Romania or Rumania or Roumania or Russia or Russian or Rwanda or Ruanda or Saint Lucia or St Lucia or Saint Vincent or St Vincent or Grenadines or Samoa or Samoan Islands or Navigator Island or Navigator Islands or Sao Tome or Senegal or Serbia or Montenegro or Seychelles or Sierra Leone or Sri Lanka or Ceylon or Solomon Islands or Somalia or Sudan or Suriname or Surinam or Swaziland or Syria or Tajikistan or Tadzhikistan or Tadjikistan or Tadzhik or Tanzania or Thailand or Togo or Togolese Republic or Tonga or Tunisia or Turkey or Turkmenistan or Turkmen or Uganda or Ukraine or USSR or Soviet Union or Union of Soviet Socialist Republics or Uzbekistan or Uzbek or Vanuatu or New Hebrides or Venezuela or Vietnam or Viet Nam or West Bank or Yemen or Yugoslavia or Zambia or Zimbabwe or Rhodesia).tw,kf

9. 4 OR 7

10. 8 AND 9
